# A synonymous mutation of *uncoupling protein 2* (*UCP2*) gene is associated with growth performance, carcass characteristics and meat quality in rabbits

**DOI:** 10.1186/s40781-016-0086-4

**Published:** 2016-01-11

**Authors:** Wen-Chao Liu, Song-Jia Lai

**Affiliations:** Farm Animal Genetic Resources Exploration and Innovation Key Laboratory of Sichuan Province, Sichuan Agricultural University, Chengdu Campus, Chengdu, 611130 China; Department of Animal Resource and Science, Dankook University, Cheonan, Choognam 330-714 South Korea

**Keywords:** *UCP2*, Growth, Carcass, Meat quality, Rabbit

## Abstract

**Background:**

Uncoupling proteins 2 (UCP2) plays an important role in energy regulation, previous studies suggested that *UCP2* is an excellent candidate gene for human obesity and growth-related traits in cattle and chicks. The current study was designed to detect the genetic variation of *UCP2* gene, and to explore the association between polymorphism of *UCP2* gene and growth, carcass and meat quality traits in rabbits.

**Results:**

A synonymous mutation in exon 1 and four variants in the first intron of the *UCP2* gene were identified by using PCR-sequencing. The synonymous mutation c.72G>A was subsequently genotyped by MassArray system (Sequenom iPLEXassay) in 248 samples from three meat rabbit breeds (94 Ira rabbits, 83 Champagne rabbits, and 71 Tianfu black rabbits). Association analysis suggested that the individuals with AA and AG genotypes showed greater 70 d body weight (*P* < 0.05), 84 d body weight (*P* < 0.01), ADG from 28 to 84 days of age (*P* < 0.05), eviscerated weight (*P* < 0.01), semi-eviscerated weight (*P* < 0.01) and semi-eviscerated slaughter percentage (*P* < 0.05), respectively. Additionally, the individuals with AA and AG genotype had a lower pH value of longissimus muscle (*P* < 0.01) and hind leg muscle (*P* < 0.05) after slaughter 24 h.

**Conclusions:**

These findings indicated that *UCP2* could be a candidate gene that associated with growth performance, body composition and meat quality in rabbits, and this would contribute to advancements in meat rabbit breeding practice.

## Background

Uncoupling proteins (UCPs) are a family of inner mitochondrial membrane transporters that dissipate the proton electrochemical gradient across mitochondrial membrane, which is believed to be accomplished via transporting protons from intermembrane to the matrix, thereby uncoupling substrate oxidation from conversion of ADP to ATP [1]. In general, UCPs reduce metabolic efficience by releasing stored energy as heat thus increasing energy expenditure, which are considered potentially important determinant of body composition [2]. Three distinct UCPs (UCP1, UCP2, and UCP3) have been identified, of which UCP2 is the principal isoform in muscular tissues and white adipose [3, 4]. Additionally, UCP2 is able to depress fatty acid (FA) synthesis in white adipose tissue [5], and UCP2 negatively regulated insulin secretion and lipogenesis was also observed [6].

Consistent with the important functions concerning energy balance and body metabolism, the *UCP2* is an excellent candidate gene for obesity-related phenotypes in human. The *UCP2* gene was mapped to human 11q13 between DS11S916 and DS11S911 markers that significantly related to obesity traits [3]. Three remarkably common variants (−866A/G, Ala55Val and 45 base-pair ins/del) in the *UCP2* gene have been widely investigated in different populations, which have been demonstrated that correlated with body weight change, BMI, lipid metabolism, resting energy expenditure, and type 2 diabetes [7–9]. In farm animals, associations of *UCP2* gene with growth performance and body composition traits have been also extensively identified. Three SNPs in bovine *UCP2* gene were in almost complete linkage disequilibrium and showed associations with body weight (BW), intramuscular fat content (IMF) and lean meat yield [10]. A synonymous mutation in exon 3 of the *UCP2* gene was linked with carcass weight (CW), and eye muscle area (EMA) in beef cattle [11]. In addition, the associations between SNPs in *UCP2* gene with BW, CW, ADG and fatness traits have been also obtained in chicks [12–14]. However, to our knowledge, the genetic polymorphisms of *UCP2* gene and association with growth-related traits in rabbit have not yet been reported. Thus, the present study was conducted to identify polymorphisms of *UCP2* gene and evaluate their effects on growth, carcass and meat quality traits in rabbits.

## Methods

Animal care and data collection procedures were approved by the Institutional Animal Care and Use Committee of Sichuan Agricultural University.

### Animals and performance traits collection

A total of 248 ear tissue samples of three meat rabbit breeds, namely, Ira rabbit (*n* = 94), Champagne rabbit (*n* = 83), Tianfu black rabbit (*n* = 71), were procured from the same farm and under the same feeding conditions. The nutritional levels and feeding management were addressed in detail in our earlier study [15]. In brief, all rabbits were fed pelleted food (16 % protein, 10.8 MJ/kg) after weaning at 28 day of age. The feed and water were provided ad libitum. Body weight was measured individually at 28 (BW28), 35 (BW35), 70 (BW70), and 84 days of age (BW84). The ADG was calculated from 28 to 84 days of age. At 84 days of age, rabbits were stunned by electro-anesthesia and slaughtered by jugulation. The carcasses were dissected according to the norms of the World Rabbit Science Association [16]. Carcass traits include eviscerated weight (EW), semi-eviscerated weight (SEW), eviscerated slaughter percentage (ESP), semi-eviscerated slaughter percentage (SESP). The EW was defined as the carcass weight that removed the head, skin, tail, front foot (below the wrist), rear (below the elbow), and all the internal organs and visceral adipose tissue. The SEW was calculated by removing the head, skin, tail, front foot (below the wrist) and rear (below the elbow), whereas retention of liver, kidney and abdominal fat. Additionally, ESP = EW/BW84; SESP = SEW/BW84. The meat quality traits include pH values of longissimus muscle after slaughter 24 h (LpH24) and right hind leg muscle after slaughter 24 h (HpH24), ripe meat ratio (RMR), intramuscular fat content of longissimus muscle (IMF-LL), and intramuscular fat content of hind leg muscle (IMF-HL). The longissimus muscle and right hind leg muscle were isolated to measure the pH values by an insert electrode pH-star (Matthäus, Pöttmes, Germany). The IMF was assessed by using Soxhlet petroleum-ether extraction.

### SNP discovery

SNPs of rabbit *UCP2* gene discovery were implemented by DNA pool sequencing, which were amplified from 40 individuals that were randomly selected from each rabbit breed. One pair of primers (Table 1) was designed to amplify exon 1 and intron 1 of *UCP2* gene based on the reference sequence (GenBank accession number NC_013669.1). The PCR amplification was carried out in a 10 μl volume containing 5 μl 2 × Taq PCR MasterMix (TIANGEN, Beijing, China), 0.4 μl of each primer, 3.2 μl ddH_2_O and 1 μl DNA template. The cycling protocol was as follows: one denaturation at 95 °C for 3 min, followed by 34 cycles of denaturation at 95 °C for 30 s, 30 s at the 58 °C, and 72 °C for 30 s, ended with final extention at 72 °C for 10 min. All PCR products were directly sequenced by Shanghai Invitrogen Biotechnology Co., Ltd (Shanghai, China). Searching mutation loci was performed using DNAstar program (DNAS Inc., Madison, WI, USA).

**Table 1:** The information of primers for PCR and MassArray

Primer	Purpose	Primer sequence (5′ → 3′)	Amplicon size (bp)	Tm (°C)
P1	Amplify exon 1	F: CTGCTTAGGGACTTGGTGCTGT	690	58.0
		R: AGCCCTTGGTGTAGAACTGTTTGA		
P2	c.72G>A genotyping	1st: ACGTTGGATGTCCTTCCTCCTGCAGGCAC	102	49.8
		2nd: ACGTTGGATGATGAGGTCATAGGTGACCAG		

### Genotyping by MassArray system

All animals were genotyped by MassArray system (Sequenom iPLEXassay, BGI Tech., Beijing, China). The MassExtend primers used in this study were listed in Table 1. In brief, the DNA samples were amplified by a multiplex PCR reaction, and then the PCR products were used for locus-specific single-base extension reaction. The resulting products were desalted and transferred to a 384-element SpectroCHIP array. The alleles were discriminated by mass spectrometry.

### Statistical analysis

The DNA sequence of *UCP2* gene were assembled and aligned for mutation analysis by using DNAstar program (DNAS Inc, Madison, WI, USA). The allele and genotype frequencies in all breeds were directly calculated. *χ*^2^ test was carried out to verify the Hardy-Weinberg equilibrium (HWE). Heterozygosity (He), effective number of alleles (Ne) and Polymorphic Information Content (PIC) were estimated based on the previous study [17]. Association of genotype with phenotype traits were analyzed by using general model (GLM) procedure of SAS 9.2 program, the *P* value <0.05 was considered significant for an association. The association with traits were used the model as follows:

$$ {\mathrm{Y}}_{\mathrm{i}\mathrm{jkl}}=\upmu +{\mathrm{S}}_{\mathrm{i}}+{\mathrm{G}}_{\mathrm{j}}+{\mathrm{B}}_{\mathrm{k}}+{\mathrm{e}}_{\mathrm{i}\mathrm{jkl}}. $$

Where: Y_ijkl_ was the trait measured on each of the ijkl animal, μ was the whole population mean, S_i_ was the fixed effect of sex, G_j_ was the fixed effect of genotype, B_k_ was the fixed effect of breeds, e_ijkl_ was the random error.

## Results

### SNP detection

In this study, sequencing was performed to investigate genetic variation in the exon1 of rabbit *UCP2* gene. By comparing the sequencing results with the rabbit *UCP2* gene sequence published in GenBank (accession number NC_013669.1), the results revealed 5 variations: a synonymous mutation and 4 intron variations. Among them, the synonymous mutation c.72G>A (Ala, GCG>GCA) was located in exon 1; the intron variations c.126+11A>G, c.126+53T>G, c.126+119A>G, c.126+128T>C were located in the first intron.

### Genetic diversity of c.72G>A in three rabbit breeds

MassArray system (Sequenom iPLEXassay) was utilized for c.72G>A genotyping in 248 samples. The frequencies of allele and genotype of the variants, as well as the population genetic indices including He, PIC, Ne are listed in Table 2. As shown in Table 2, three different genotypes (AA, AG and GG) were identified and with similar frequency of allele and genotype in the analyzed three breeds, the heterozygous AG was a major genotype in these breeds (ranging from 0.51 in Ira to 0.60 in Champagne and then 0.63 in Tianfu black). Allelic frequencies for the G allele ranged from 0.38 in Ira to 0.43 in Champagne, and 0.51 in Tianfu black. As described in Table 2, the gene heterozygosity (He) varied from 0.47 (Ira) to 0.50 (Tianfu black), while the effective allele number (Ne) ranged from 1.90 (Ira) to 1.99 (Tianfu black). Based on the classification of PIC values (low polymorphism if PIC value <0.25, moderate polymorphism if 0.25<PIC<0.50, and high polymorphism if PIC>0.50) [17], the c.72G>A loci possessed medium genetic diversity (0.25<PIC<0.5) in the analyzed breeds and implied this loci have a relatively large selection of potential. Additionally, the c.72G>A loci was at Hardy-Weinberg disequilibrium (*P* < 0.05 or *P* < 0.01) in three rabbit breeds, implying that rapid, powerful, and effective selection strategies might change the allelic balance.

**Table 2:** The frequencies of allele and genotype of the c.72G>A loci

Breeds (*n*)	Genotype frequency (*n*)			Allele frequency		Genetic characteristic			*x* ^2^	*P*-value
	AA	AG	GG	A	G	He	PIC	Ne		
Ira (94)	0.36 (34)	0.51 (48)	0.13 (12)	0.62	0.38	0.47	0.36	1.90	0.61	>0.05
Champagne (83)	0.27 (22)	0.60 (50)	0.13 (11)	0.57	0.43	0.49	0.37	1.97	4.25	<0.05
Tianfu (71)	0.17 (12)	0.63 (45)	0.20 (14)	0.49	0.51	0.50	0.37	1.99	5.12	<0.05
Total (248)	0.27 (68)	0.58 (143)	0.15 (37)	0.56	0.44	0.49	0.37	1.99	7.30	<0.01

*He* heterozygosity, *PIC* polymorphism information content, *Ne* effective number of alleles

*x* ^2^ Hardy-Weinberg equilibrium *x* ^2^ value. *x* ^2^_0.05_(df = 1) = 3.84, *x* ^2^_0.01_(df = 1) = 6.63

### Association of c.72G>A in *UCP2* gene with phenotypic traits in rabbits

Data in Table 3 showed the associations of c.72G>A with growth parameters in rabbits. There were significant associations between this SNP and eight traits in all tested rabbit breeds, namely, BW70 (*P* = 0.013), BW84 (*P* = 0.008), ADG (*P* = 0.029), EW (*P* = 0.001), SEW (*P* = 0.001), SESP (*P* = 0.037), LpH24 (*P* = 0.002) and HpH24 (*P* = 0.028). Furthermore, individuals with genotypes AA and AG demonstrated significantly superior growth performance and carcass traits when compared with those with genotype GG, which reflects that the A allele was responsible for the observed positive effects on growth and carcass traits.

**Table 3:** Association between c.72G>A in *UCP2* gene with growth, carcass and meat quality traits in rabbit

Traits^1^	Genotypes			*P*-value*
	AA (*n* = 68)	AG (*n* = 143)	GG (*n* = 37)	
BW28 (g)	523.00 ± 12.09	534.01 ± 8.35	517.62 ± 14.15	0.538
BW35 (g)	858.36 ± 35.30	809.28 ± 20.84	794.16 ± 30.61	0.357
BW70 (g)	2208.50 ± 21.62^A^	2183.85 ± 14.66^A^	2130.99 ± 24.73^B^	**0.013**
BW84 (g)	2568.17 ± 17.84^A^	2533.93 ± 26.32^A^	2495.61 ± 20.41^B^	**0.008**
ADG (g)	36.04 ± 0.38^a^	35.97 ± 0.65^a^	32.82 ± 0.57^b^	**0.029**
EW (g)	1333.85 ± 15.60^A^	1330.53 ± 11.39^A^	1248.37 ± 20.16^B^	**0.001**
ESP (%)	0.5261 ± 0.0025	0.5271 ± 0.0018	0.5197 ± 0.0032	0.134
SEW (g)	1445.35 ± 16.64^A^	1439.45 ± 12.16^A^	1348.04 ± 21.51^B^	**0.001**
SESP (%)	0.5700 ± 0.0025^a^	0.5702 ± 0.0018^a^	0.5612 ± 0.0032^b^	**0.037**
LpH24	5.72 ± 0.02^A^	5.73 ± 0.01^A^	5.81 ± 0.02^B^	**0.002**
HpH24	5.74 ± 0.01^a^	5.73 ± 0.02^a^	5.80 ± 0.01^b^	**0.028**
RMR (%)	0.67 ± 0.0076	0.67 ± 0.0055	0.65 ± 0.0099	0.110
IMF-HL (%)	0.0043 ± 0.0004	0.0046 ± 0.0003	0.0048 ± 0.0006	0.737
IMF-LL (%)	0.0067 ± 0.0010	0.0077 ± 0.0007	0.0084 ± 0.0012	0.518

Values are presented by the least squares means ± standard error

^a^ *BW28*, *BW35*, *BW70*, and *BW84* body weight of 28, 35, 70 and 84 days of age, respectively, *ADG* average daily gain from 28 to 84 days of age, *EW* eviscerated weight, *ESP* eviscerated slaughter percentage, *SEW* semi-eviscerated weight, *SESP* semi-eviscerated slaughter percentage, *LpH24* pH of longissimus muscle after slaughter 24 h, *HpH24* pH of hind leg muscle after slaughter 24 h, *IMF*-*LL* intramuscular fat content of longissimus muscle, *IMF*-*HL* intramuscular fat content of hind leg muscle

*P-value: Overall significance value for an effect of the genotype, the boldface reflects the *P* value less than 0.05. Values with different letters (a, b and A, B) within the same row differ significantly at *P* < 0.05 and *P* < 0.01, respectively

## Discussion

Variations in energy expenditure could be responsible for differences in metabolic rate among individuals, also, may be one of the underlying sources of body weight change [18]. The uncoupling protein plays a key role in burning calories and generating heat by creating a pathway that allows dissipation of the proton electrochemical gradient across the inner mitochondrial membrane, without coupling to any other energy-consuming process. In addition, this pathway has been demonstrated in the regulation of energy expenditure, body composition and and glucose metabolism [19]. Likewise, in the present study, the association study revealed significant relationships between the c.72G>A loci and BW70, BW84, ADG, EW, SEW and SESP, respectively, suggested that the *UCP2* gene had positive effects on growth and carcass traits. This is consistent with the pivotal function of UCP2 regulating energy expenditure and body composition. In addition, another important function of UCP2 was to affect insulin secretion and glucose metabolism [4], thus involved in the body growth and development. Which can explain the significant association results between *UCP2* gene polymorphisms with growth and carcass traits in this study. Also, in agreement with our results, Cassell et al. [7] reported that the polymorphisms of *UCP2* gene influenced the body wight gain in Indian subjects. Lee et al. [9] suggested that variants in *UCP2* gene related to BMI in Korean population. Similarly, the relationship between polymorphisms of *UCP2* gene and growth-related traits in beef cattle were observed by Sherman et al. [10] and Ryu et al. [11], who demonstrated that the synonymous mutations in *UCP2* gene were significantly associated with body wight, feed intake and carcass weight. Besides, it should be noted that individuals with genotype AA and AG had superior performance compared to those with genotype GG, this suggested that the A allele was responsible for the observed positive effects on growth and carcass traits. Therefore, it can be concluded that the A allele of c.72G>A should be considered and incorporated into a panel of markers for rabbit breeding.

In addition to growth and carcass traits, the current study also demonstrated that the c.72G>A was significantly linked with the pH value of longissimus muscle and hind leg muscle after slaughter 24 h. It is well known that pH is an important characteristic of meat quality and affects the water holding capacity of meat [20]. And with the passage of storage time after slaughter, the muscle cells still carry out a variety of biochemical reactions and glycolysis generates a lot of lactic acid, resulting in decreased pH [21]. To the best of our knowledge, there is relatively limited data on the effect of *UCP2* gene on meat pH, thus, no comparisons could be made with other studies. And further studies will be necessary to determine whether the *UCP2* gene involved in the biochemical processes of glycolysis. Interestingly, this study failed to reveal any significant association of c.72G>A with IMF-HL and IMF-LL, this is inconsistent with the active role of UCP2 in adipose oxidative metabolism. Adipose oxidative metabolism would presumably increases the production of reactive oxygen species (ROS), and it is believed that the uncoupling activity of UCP2 is physiologically important in modulating the generation of ROS from mitochondria of certain cell types [22]. Additionally, the association of *UCP2* gene and eye muscle area was observed in beef cattle by Ryu et al. [11]. It is postulated that the contradictory results may be due to the less body fat content in rabbit, so the effect of rabbit *UCP2* gene on adipose oxidative metabolism is relatively slight.

It is remarkable that the associated SNP (c.72G>A) is a synonymous mutation and did not cause amino acid change. Presumably, the synonymous mutation might result in codon usage bias, thus influence gene transcriptional efficiency and/or stability of mRNA, as well as protein advanced structure. Actually, several previous studies revealed that the synonymous mutation associated with traits by affecting gene expression in rabbits. For instance, a synonymous mutation in *NOD2* and *NLRP3* had lead to the gene itself expression change and related to rabbit digestive disorders [23, 24]. Therefore, the significantly association in the current study may have resulted from: 1) the synonymous mutation c.72G>A produce codon usage bias and affect gene transcription; 2) the c.72G>A may be associated with the variants in intron regions and have positive effects on transcription factor binding or mRNA splicing, thus influencing the expression of the gene itself; 3) the c.72G>A loci may be in linkage disequilibrium with other causative variants in promoter regions, as well as other genes on the same chromosome that have a practical effect on these performance traits. However, further studies are essential to confirm these hypotheses.

## Conclusion

In summary, the current study revealed that the synonymous mutation c.72G>A of *UCP2* gene was significantly associated with growth performance, carcass traits and meat pH, this would contribute to implementing the marker-assisted selection in breeding and genetics in meat rabbit. Further researches will be needed to confirm the associated effect on other population and breeds.
